# The (Still Unknown) Hypothetical Protective Role of COVID-19 Therapy in Bladder Cancer

**DOI:** 10.3390/jcm10235473

**Published:** 2021-11-23

**Authors:** Luca Di Gianfrancesco

**Affiliations:** 1 Department of Oncological Urology, Istituto Oncologico Veneto, Castelfranco Veneto, 31033 Treviso, Italy; luca.digian@libero.it or luca.digianfrancesco@iov.veneto.it; Tel.: +39-320-216-7165; 2 Department of Urology, Università Cattolica del Sacro Cuore di Roma, 00168 Roma, Italy

**Keywords:** COVID-19, bladder cancer, repurposing/repositioning modality, protective role, treatment

## Abstract

The COVID-19 pandemic continues to put a strain on the entire world population. The common features of bladder cancer (BCa) and COVID infection have been widely reported and discussion may continue regarding treatment as well. We have highlighted how COVID-19 therapy has many implications with BCa therapy, in particular with potential protective role.

## 1. Introduction

The COVID-19 pandemic continues to put a strain on the entire world population and translational efforts concern all health professionals, and the limited time available made it necessary to use specific drugs for other diseases in a drug repurposing/repositioning modality.

The common features of bladder cancer (BCa) and COVID-19 infection were exhaustively reported, both in predisposing factors and in clinical-pathological terms [1]. Based on this evidence, an association deserves to be also evaluated also in terms of therapy and how it might impact on the BCa.

The hypothesis of this study consisted in the translation of the evidence on the common clinical-pathological characteristics of the two diseases to the treatment issue. The aim of this study was to underline how the COVID-19 therapy presents many potential implications with BCa therapy.

## 2. Materials and Methods

The PubMed, Medline, Web of Science databases were searched in order to identify all the related reports discussing the COVID-19 therapy and the BCa, by using and combining the terms “bladder cancer”, “urothelial carcinoma”, “urothelial cancer”, “COVID-19”, “SARS-CoV-2”, “therapy”, “FDA (Food and Drug Administration)”, “EMA (European Medicines Agency)”, and “EUA (Emergency Use Authorization)”. Firstly, the research focused on the drugs employed against COVID-19 infection, then those drugs with a link to bladder cancer were fully considered in this evaluation.

There were no date or language restrictions on the research in order to establish relevant references for link between given drug and bladder cancer.

Due to the nature of this manuscript our study is institutional Review board (IRB) exempt and the informed consent was not applicable.

The primary endpoint was to identify drugs with a clinical implication in both the two considered pathologies (COVID-19 infection and BCa). The secondary endpoint was to describe the mechanism of action and the targets of these drugs in order to detect the link between the treatments of the two diseases.

## 3. Results

Evidence Synthesis

Among the treatments for COVID-19 infection used in repurposing/repositioning modalities with applicability in the BCa treatment, we identified: Chloroquine and derivatives, Lopinavir/Rotinavir, Ribavirin, Remdesivir, Tocilizumab, Corticosteroids, Heparin, Bacillus of Calmette-Guerin (BCG) (Table 1).

## 4. Discussion

### 4.1. Chloroquine and Derivatives

Chloroquine and derivatives have been used for years for the treatment of malaria and chronic inflammatory diseases such as lupus erythematosus and rheumatoid arthritis. These drugs block the entry of the pathogen into the cells, and they have immunomodulatory effects through the attenuation of the production of cytokines and the inhibition of the autophagy and lysosomal activities of the host cells.

Chloroquine and derivatives inhibit the fusion of severe acute respiratory syndrome coronavirus 2 (SARS-CoV-2) and the host cell membranes [16]. Chloroquine inhibits glycosylation of the cellular angiotensin-converting enzyme 2 receptor, which may interfere with binding of SARSCoV to the cell receptor [17], and they block the transport of SARS-CoV-2 from early endosomes to endolysosomes, possibly preventing the release of the viral genome. Despite the FDA revoked the EUA, the results are promising in terms of improving the radiological picture, better virus clearance and disease progression.

Regarding BCa, Lin et al. showed the potential anticancer properties of chloroquine by inhibiting the proliferation of multiple human bladder cell lines; the BCa cells treated with chloroquine or derivatives underwent a greater level of autophagy and apoptosis [2]. Chen et al. showed that chloroquine-induced lysosomal membrane permeability increases apoptosis levels leading to BCa cell mote [18] and Wang et al. demonstrated how this agent increases the radiosensitivity of BCa cells through the inhibition of autophagy and the activation of apoptosis of the same cells [3].

### 4.2. Lopinavir/Ritonavir

Lopinavir/Ritonavir is an FDA-approved combination for the HIV-treatment and demonstrated in vitro activity against coronavirus through the inhibition of 3-chymotrypsin-like protease. The evidence in COVID-treatment is not so strong but efficacy in terms of reduced mortality and intubation rates has nevertheless been recorded. The combinations of ritonavir with agents that increase the number of unfolded proteins induce endoplasmic reticulum stress cooperatively and thereby kill cancer cells effectively. Moreover, Ritonavir increases the intracellular concentration of combined drugs by inhibiting their degradation and efflux from cancer cells and thereby enhancing their antineoplastic activity. Furthermore, ritonavir’s antineoplastic activity includes modulation of immune system activity [5].

Okubo et al. explored the new approach to the cancer treatment by the induction of the endoplasmic reticulum: Lopinavir/Ritonavir inhibited synergistically the growth of BCa cells through the induction of stress in the endoplasmic reticulum with: increased expression of the AMP-activated protein kinase, suppression of the mammalian target of rapamycin pathway, increased expression of tumor necrosis factor-related apoptosis-inducing ligand (TRAIL) and a sensitization of neoplastic cells to TRAIL [4].

### 4.3. Ribavirin

This drug is a guanosine analogue and RNA-synthesis inhibitor employed in the therapy chronic hepatitis C virus (HCV) infection.

Ribavirin is endowed with COVID-19 antiRNA-dependent RNA polymerase (RdRp) activity. However, its in vitro activity against SARS-CoV was limited and required high concentrations and combination therapy to inhibit viral replication. It was demonstrated that ribavirin inhibits inosine 5′-phosphate dehydrogenase (IMPDH), the first enzyme specific for the de novo synthesis of guanosine monophosphate (GMP) [19]; the IMPDH is necessary for DNA and RNA synthesis, and it has been linked to cell growth, differentiation and malignant transformation [20,21].

Regarding BCa, many efforts focused on RNA pathways regulation and, as in prostate cancer (PCa), this agent deserves the opportunity to explore its anticancer activity even in urothelial tumor. The treatment of PCa-cells with ribavirin decreased their resistance against treatment with docetaxel: this indicated that ribavirin reversed the gene expression, including that of humoral factors, in the OCT4-expressing PCa cells selected using the EOS system [22], and this deserves to be explored even in Bca.

### 4.4. Remdesivir/Gemcitabine

Remdesivir has a broad spectrum of antiviral activities against RNA viruses, including SARSCoV-1. It selectively inhibits virus replication by targeting its viral RNA-dependent RNApolymerases (RdRp).

Remdesivir received FDA- and EMA-approval for COVID-infection. Remdesivir is a pro-drug, its active analogue enters and accumulates in cells, inhibiting viral RdRp [23] and stopping viral replication. Coronaviruses have a “proofreading” enzyme (exoribonuclease) that corrects errors in the RNA sequence, potentially limiting the effects of analogues [24,25], but remdesivir is able to evade this proofreading [26,27].

This compound is not cancer-related. The main similar drug, in a speculative view, is represented by gemcitabine, a cytidine analog FDA-approved for the treatment of various cancers [28] and with an effective broad-spectrum antiviral activity against multiple RNA viruses [29]. Gemcitabine is one of the most commonly used analog nucleoside as chemotherapy, sistemically and intravesical administered, in BCa [30].

In a speculative way, Remdesivir might exert its potential protective role on BCa, and Gemcitabine on COVID-19. The wide and recommended employment of the drugs might provide a large amount of patients to be observed.

### 4.5. Tocilizumab

Tocilizumab is an inhibitor of interleukin (IL) 6 approved for the treatment of rheumatoid arthritis, giant cell arteritis, and cytokine release syndrome during chimeric antigen receptor T cell therapy (CAR-T). IL-6 is an inflammatory cytokine with effects on lymphocytes, by inducing acute phase reactants such as C-reactive protein (CRP), fibrinogen and hepcidin, and promotes CD4 T-helper 17 and CD8 cytotoxic T-cell differentiation and antibody production. Moreover, IL-6 plays an important role in controlling viral infections such as influenza A, SARS-CoV-1, and herpesvirus.

During COVID-19 infection, an increased level of IL-6 and CRP is correlated with disease severity and mortality. Thus, the blockage of IL-6 activity might play a role in mitigating the inflammatory response and, subsequently, it might improve clinical outcomes in patients with COVID-19 [31]. Several RCTs of tocilizumab, alone or in combination, in patients with COVID-19 with severe pneumonia are ongoing in China, and it is included in the current Chinese national treatment guidelines. Tocilizumab in COVID-19 patients reduce their stay in hospital and improve clinical outcomes [32,33,34].

When combined with conventional drugs, Tocilizumab provides promising results for treating cancer. As for cancer, the IL-6-signaling pathway represents an attractive target for therapeutic or preventive interventions even for infectious diseases and chronic inflammatory diseases. In the BCa specimens, the IL-6 was overexpressed compared to nonmalignant tissues at both mRNA and protein levels; moreover, higher clinical stage, higher recurrence rate, and reduced survival rate were correlated to positive IL-6 staining, and when IL-6 was blocked, the tumor growth and invasive capability were attenuated. A decreased cell proliferation, a less epithelial-mesenchymal transition (EMT), a decreased DNA methyltransferase-1 expression and an attenuated angiogenesis were the underlying changes included. Therefore, targeting IL-6 may be a promising strategy for treating bladder cancer [35].

Moreover, this agent is under consideration in a study evaluating the efficacy and safety of multiple immunotherapy-based treatment combinations in patients with locally advanced or metastatic urothelial carcinoma after failure with platinum-containing chemotherapy [10].

### 4.6. Corticosteroids

Corticosteroids are a class of steroid-hormones released by the adrenal cortex, and the term is generally used to refer to glucocorticoids. Named for their effect in carbohydrate metabolism, glucocorticoids regulate several cellular functions including development, homeostasis, metabolism, cognition, and inflammation. Due to their profound immune-modulatory actions, glucocorticoids have become a clinical mainstay for the treatment of numerous inflammatory and autoimmune diseases. Unfortunately, the therapeutic benefits of glucocorticoids are limited by the adverse side effects that are associated with high dose and long-term use [36]. Waterhouse et al. highlighted the need of a risk-benefit analysis of corticosteroids use for each individual patient undergoing urological cancer treatment, and it should be assessed whether the theoretical risks associated with steroids and COVID-19 interfere with the clear cancer management benefits of systemic cancer therapy and corticosteroids [37].

Regarding BCa, in the study of Ide et al. the activities of glucocorticoid-receptor appeared to be significantly correlated to cancer, even in term of prediction of prognosis [12]. In the study of Zheng et al., the activation of glucocorticoid-receptor lead to the promotion of cell proliferation via inhibiting apoptosis yet repression of cell invasion and metastasis. These results might pave the way for the development of improved chemotherapy regimens, including or excluding glucocorticoid receptor agonists/antagonists, for urothelial carcinoma [13].

### 4.7. Heparin

Prior evidence showed that low molecular weight heparin (LMWH) has anti-inflammatory properties and reduces the biological activity and levels of IL-6 [38]. The heparin anti-COVID mechanisms of action lead to slowing of inflammatory response, lower levels of IL-6, higher lymphocyte levels, and less coagulopathy. The use of heparin lead to a lower mortality rate among patients with elevated sepsis-induced coagulopathy score and D-dimer.

In the field of BCa, Selectins were demonstrated to be cell-adhesion molecules mediating attachment of leukocytes to activated endothelium as well as the adhesion reaction of tumors during malignancy. Heparin, which is known to attenuate metastasis, is a potent blocker of selectins. Here, the role of selectins in metastasis and the potential of heparin to modulate malignancy [14].

### 4.8. Bacillus of Calmette-Guerin (BCG)

The innate immune system can develop “memory” (trained immunity), through epigenetic reprogramming of different innate immune cell types. In both human and murine models BCG-vaccination lead to trained immunity, which helps eliminate various non-mycobacterium infections including staphylococci, candidiasis, yellow fever, and influenza.

Based on data from some epidemiological studies, there are some current clinical trials on the use BCG as a possible prophylactic vaccine against SARS-CoV-2 [39].

BCG is one of the most widely used drugs in urology, as a standard-of-care therapy for high-risk non-muscle invasive bladder cancer (HR-NMIBC). The issue of intravesical-BCG during the COVID-19 pandemic has been widely explored, and panels of experts suggested to continue with the therapy due to its role as reference adjuvant treatment for HR-NMIBC. To date, there are no reports that patients receiving intravesical BCG have a higher risk of contracting COVID-19. In addition, intravesical BCG also exerts a systemic immunologic effect, that might be protective to this subgroup of urological patients [40,41,42,43].

### 4.9. Limitations

The limitations of the present study were: mere speculative correlation between BCa and COVID therapies based only on previous findings on pathological mechanism of actions of the two diseases and not on rigorous researches; the evidence of anti-COVID efficacy of most drugs was questionable and based on theoretical speculations; the lack of acknowledgement and rigorous assessment on how frequently the COVID-drugs were or will be used in daily practice; some drugs were evaluated only in vitro experiments yet; the lack of statistical comparison between evidences due to the speculative nature of the study.

### 4.10. New Perspectives

The research stimulated by these considerations might lead to some compelling perspectives: some treatments might have true benefit and possible trial establishment; there are many insightful reasons for the urologists to learn more about these treatments: the opportunity to follow-up patients with proven diagnosis of BCa and undergoing (intravesical or systemic) treatments for BCa; the basis for observational trials focusing on the potential lower incidence of Bca and with lower stage at presentation (due to the protective role of some announced treatment on BCa). The main take-home message of the study is: COVID-19 infection forced to use drugs in a repurposing/repositioning modality with some potential benefits to BCa patients and the urologist should pay full attention to this issue.

## 5. Conclusions

The similarities between COVID-infection and BCa might concern also the therapy; many drugs used against COVID have already been surprisingly evaluated in the context of BCa, and with curative and even protective effects. Further evidence based on long follow-up are needed to consolidate these hypotheses; to date, no ongoing trial is focusing on the clinic-pathological impact of the COVID-therapy on BCa. The conclusions are surely speculative, but the follow-up of this issue could be very insightful.

## Figures and Tables

**Table 1:** Treatments for COVID-19 infection used in repurposing/repositioning modalities with applicability in the BCa treatment.

Drug	Principal Mechanism of Action	COVID		Bladder Cancer	
		Mechanism of Action for COVID Treatment	Approval	Possible Mechanism of Action	Supporting Data
Chloriquine and derivatives	Inhibition of lysosomal activity and autophagy (autoimmune diseases)	block the entry of the virus into the cells by inhibiting glycosylation of the host receptors, proteolytic processes and endosomal acidification; attenuation of the production of cytokines and the inhibition of the autophagy and lysosomal activities of the host cells	EUA revoked by FDA	inhibiting the proliferation of multiple human bladder cell lines;induced lysosomal membrane permeability increases apoptosis levels leading to bladder cancer cell mote	in vitro [2] in vitro [3]
Lopinavir/Rotinavir	Protease inhibitors (HIV infection)	inhibition of 3-chymotrypsin-like protease	-	inhibition of the growth of bladder cancer by stress-induction in endoplasmic reticulum: increased expression of the AMP-activated protein kinase, suppression of the mammalian target of rapamycin pathway, increased expression of TRAIL, sensitization of neoplastic cells to TRAIL; Modulation of immune system activity	in vitro [4] in vitro [5]
Ribavirin	guanosine analogue and RNA synthesis inhibitor (HCV infection)	endowed with COVID-19 antiRNA dependent RNA polymerase (RdRp) activity	-	RNA pathways regulationInhibition of the activities of the eIF4E	Speculative [6] Speculative [7]
Remdesivir	nucleotide analogue with a proved mechanism of action as an inhibitor of RNA-dependent RNA polymerases (activity against SARS-CoV-1, MERS-CoV)	CoV inhibition through RNA-dependent RNA polymerase (RdRp)-mediated mechanism	FDA and EMA	inhibitory activity on the pyrimidine biosynthesisinhibition of sustained protein synthesis and is therefore a fundamental determinant of the capacity of a cell to grow	Speculative [8]
Gemcitabine	cytidine analog (treatment of non-small cell lung cancer, pancreatic cancer, bladder cancer and breast cancer)	Inhibition of SARS-CoV-2 replication through the modulation of nucleotide biosynthesis in vitro	-	inhibitor of ribonucleotide reductase, inhibitor of DNA synthesis and repair, inhibition of the growth of several cell lines	in vivo [9]
Tocilizumab	anti-IL-6 (rheumatoid arthritis, giant cell arteritis, cytokine release syndrome during CAR-T)	blocking interleukin 6 activity mitigates the inflammatory response	-	Decreased cell proliferation, less EMT, decreased DNA methyltransferase 1 expression and attenuated angiogenesis; Tumor growth and invasive capability attenuated with blockage of IL-6	in vivo—ongoing trial [10] in vitro [11]
Corticosteroids	Regulation of the expression of anti-inflammatory proteins in nucleus, repression of the expression of proinflammatory proteins in cytosol, prevention of the translocation of transcription factors from cytosol into nucleus. (inflammatory and autoimmune diseases)	Decrease of the host inflammatory responses in the lungs	-	the glucocorticoid-receptor activation resulted in promotion of cell proliferation via inhibiting apoptosis yet repression of cell invasion and metastasis	in vitro [12,13]
Covalescent plasma	IgG replacement, anti-inflammatory and immunomodulatory effect (replacement for Primary Immunodeficiency Diseases, autoimmune or auto-inflammatory conditions)	Antibodies against SARS-CoV-2 with suppression of the virus and the modification of the inflammatory response	FDA issued EUA for the use in hospitalized patients	-	Speculative
Heparin	anti-inflammatory properties and reduces the biological activity and levels of IL-6 (acute coronary syndrome, atrial fibrillation, deep-vein thrombosis, pulmonary embolism, hemofiltration)	slowing of inflammatory response, lower levels of IL-6, higher lymphocyte levels, and less coagulopathy	-	Block of selectins, cell adhesion molecules mediating attachment of leukocytes to activated endothelium as well as the adhesion reaction of tumors during malignancy and metastasis	Speculative [14]
BCG (systemic administration)	epigenetic reprogramming and induction of trained immunity (prevention of tuberculosis)	Decrease in “cytokine storm”; stimulation of TH1 response; reduction in TH2 response	-	Development of innate immunity and resident memory T cells. The preexisting BCG-specific T cells improve intravesical immunotherapy for bladder cancer	in vivo [15]

FDA: Food and Drug Administration; EMA: European Medicines Agency; EUA: Emergency Use Authorization; TRAIL: TNF-related apoptosis-inducing ligand; eIF4E: eukaryotic translation initiation factor; EMT: epithelial-mesenchymal transition; IL: interleukin; Ig: immunoglobulin; CAR-T: chimeric antigen receptor T cell therapy; TH: T helper cell.

## Data Availability

Not applicable.
